# Supplementary material for: Pyruvate Dehydrogenase Kinase 1 inhibition mediated oxidative phosphorylation enhancement in cartilage promotes osteoarthritis progression
Source: BMC Musculoskelet Disord. 2023 Jul 20;24:597. doi: 10.1186/s12891-023-06585-6 (PMC10357736; doi:10.1186/s12891-023-06585-6)
Supplement: Supplementary file 1 — Additional file 1. [file 12891_2023_6585_MOESM1_ESM.zip › BP.Gsea.1653623667859/GOBP_I_KAPPAB_PHOSPHORYLATION.html]

Details for gene set GOBP\_I\_KAPPAB\_PHOSPHORYLATION[GSEA]

|  || Dataset | 55457\_collapsed\_to\_symbols.cls.cls #OA\_versus\_normal.cls.cls #OA\_versus\_normal\_repos |
| Phenotype | cls.cls#OA\_versus\_normal\_repos |
| Upregulated in class | OA |
| GeneSet | GOBP\_I\_KAPPAB\_PHOSPHORYLATION |
| Enrichment Score (ES) | 0.5877387 |
| Normalized Enrichment Score (NES) | 1.8815025 |
| Nominal p-value | 0.0023419203 |
| FDR q-value | 0.04771451 |
| FWER p-Value | 0.819 |
Table: GSEA Results Summary

  

Fig 1: Enrichment plot: GOBP\_I\_KAPPAB\_PHOSPHORYLATION      
 Profile of the Running ES Score & Positions of GeneSet Members on the Rank Ordered List

  

| SYMBOL | TITLE | RANK IN GENE LIST | RANK METRIC SCORE | RUNNING ES | CORE ENRICHMENT || 1 | TLR7 | toll like receptor 7 [Source:HGNC Symbol;Acc:HGNC:15631] | 61 | 0.971 | 0.1306 | Yes |
| 2 | PRDX4 | peroxiredoxin 4 [Source:HGNC Symbol;Acc:HGNC:17169] | 68 | 0.959 | 0.2638 | Yes |
| 3 | TLR3 | toll like receptor 3 [Source:HGNC Symbol;Acc:HGNC:11849] | 175 | 0.837 | 0.3723 | Yes |
| 4 | CX3CR1 | C-X3-C motif chemokine receptor 1 [Source:HGNC Symbol;Acc:HGNC:2558] | 230 | 0.799 | 0.4795 | Yes |
| 5 | PLCG2 | phospholipase C gamma 2 [Source:HGNC Symbol;Acc:HGNC:9066] | 725 | 0.588 | 0.5231 | Yes |
| 6 | AKT1 | AKT serine/threonine kinase 1 [Source:HGNC Symbol;Acc:HGNC:391] | 1192 | 0.478 | 0.5536 | Yes |
| 7 | MAP3K7 | mitogen-activated protein kinase kinase kinase 7 [Source:HGNC Symbol;Acc:HGNC:6859] | 1701 | 0.399 | 0.5697 | Yes |
| 8 | TNF | tumor necrosis factor [Source:HGNC Symbol;Acc:HGNC:11892] | 2098 | 0.350 | 0.5877 | Yes |
| 9 | SIRPA | signal regulatory protein alpha [Source:HGNC Symbol;Acc:HGNC:9662] | 3599 | 0.193 | 0.4983 | No |
| 10 | CX3CL1 | C-X3-C motif chemokine ligand 1 [Source:HGNC Symbol;Acc:HGNC:10647] | 5764 | 0.011 | 0.3318 | No |
| 11 | IKBKB | inhibitor of nuclear factor kappa B kinase subunit beta [Source:HGNC Symbol;Acc:HGNC:5960] | 5914 | 0.001 | 0.3204 | No |
| 12 | ERC1 | ELKS/RAB6-interacting/CAST family member 1 [Source:HGNC Symbol;Acc:HGNC:17072] | 6147 | -0.016 | 0.3046 | No |
| 13 | TLR4 | toll like receptor 4 [Source:HGNC Symbol;Acc:HGNC:11850] | 7703 | -0.134 | 0.2027 | No |
| 14 | TLR2 | toll like receptor 2 [Source:HGNC Symbol;Acc:HGNC:11848] | 7991 | -0.154 | 0.2019 | No |
| 15 | TRAF2 | TNF receptor associated factor 2 [Source:HGNC Symbol;Acc:HGNC:12032] | 8322 | -0.180 | 0.2013 | No |
| 16 | IKBKE | inhibitor of nuclear factor kappa B kinase subunit epsilon [Source:HGNC Symbol;Acc:HGNC:14552] | 9646 | -0.287 | 0.1386 | No |
| 17 | DDRGK1 | DDRGK domain containing 1 [Source:HGNC Symbol;Acc:HGNC:16110] | 9741 | -0.296 | 0.1725 | No |
| 18 | CHUK | component of inhibitor of nuclear factor kappa B kinase complex [Source:HGNC Symbol;Acc:HGNC:1974] | 11624 | -0.521 | 0.0990 | No |
Table: GSEA details [plain text format]

  

Fig 2: GOBP\_I\_KAPPAB\_PHOSPHORYLATION      
 Blue-Pink O' Gram in the Space of the Analyzed GeneSet

  

Fig 3: GOBP\_I\_KAPPAB\_PHOSPHORYLATION: Random ES distribution      
 Gene set null distribution of ES for **GOBP\_I\_KAPPAB\_PHOSPHORYLATION**
